# The energetic behaviour of the human foot across a range of running speeds

**DOI:** 10.1038/s41598-018-28946-1

**Published:** 2018-07-12

**Authors:** Luke A. Kelly, Andrew G. Cresswell, Dominic J. Farris

**Affiliations:** 1 School of Human Movement and Nutrition Sciences, The University of Queensland, Brisbane, Australia; 2 Sport and Health Sciences, University of Exeter, Exeter, United Kingdom

## Abstract

The human foot contains passive elastic tissues that have spring-like qualities, storing and returning mechanical energy and other tissues that behave as dampers, dissipating energy. Additionally the intrinsic and extrinsic foot muscles have the capacity to act as dampers and motors, dissipating and generating mechanical energy. It remains unknown as to how the contribution of all passive and active tissues combine to produce the overall energetic function of the foot during running. Therefore, the aim of this study was to determine if the foot behaves globally as an active spring-damper during running. Fourteen participants ran on a force-instrumented treadmill at 2.2 ms^−1^, 3.3 ms^−1^ and 4.4 ms^−1^, while foot segment motion was collected simultaneously with kinetic measurements. A unified deformable segment model was applied to quantify the instantaneous power of the foot segment during ground contact and mechanical work was calculated by integrating the foot power data. At all running speeds, the foot absorbed energy from early stance through to mid-stance and subsequently returned/generated a proportion of this energy in late stance. The magnitude of negative work performed increased with running speed, while the magnitude of positive work remained relatively constant across all running speeds. The proportion of energy dissipated relative to that absorbed (foot dissipation-ratio) was always greater than zero and increased with running speed, suggesting that the foot behaves as a viscous spring-damper.

## Introduction

The human foot is often described as possessing spring-like qualities during running^1–3^. The longitudinal arch (LA) of the foot lengthens and lowers (arch compression) during early stance, allowing mechanical energy to be stored in the stretched elastic ligaments and tendons that span this structure^3^. In late stance elastic recoil of these tissues causes the LA to rise and shorten (arch recoil) with stored mechanical energy being returned to the body, contributing positive power for forward propulsion^1,3–5^. This mechanism is considered a key contributor to human running economy, delivering an estimated 8–17% of the mechanical energy required for each step^1,3^.

Despite the proposed energy conservation mechanisms within the foot, there are also structures in the foot that dissipate energy. The plantar fat pads of the heel and forefoot are fibro-adipose structures that provide protection against localised peak bone stresses^6^. The plantar fat pads deform as the foot collides with the ground, absorbing a portion of the mechanical energy associated with foot-ground contact^7–9^. Approximately 20–50% of the energy absorbed within these structures is dissipated^8,10,11^ in a velocity dependent manner, with a greater percentage of absorbed energy being dissipated at higher impact velocities^7,9,12^, much like a visco-elastic damper.

The plantar intrinsic foot muscles are a group of muscles located within the foot, spanning a similar anatomical pathway to the Plantar Fascia (PF)^13,14^. The muscle tendon units (MTUs) of these muscles actively lengthen during early stance, thus they have the capacity to dissipate mechanical energy, while active shorting of the MTUs in late stance may generate mechanical energy to aid propulsion^4^. Similarly the tibialis posterior muscle has been shown to absorb and return mechanical energy at the hind-foot joints, via stretch and recoil of its long elastic tendon^15,16^.

It is apparent that individual soft tissue structures within the foot may act to recycle, dissipate and generate mechanical energy. However, it remains unknown as to how the contribution of the many deformable structures within the foot combine, and what the overall energetic function of the foot is during running. A greater understanding of this function is required to further inform development of biologically inspired prosthesis and wearable assistive devices. Therefore, the aim of this study was to determine the global energetic profile of the foot during running at various speeds. Using a novel approach to quantify mechanical work performed at the foot, we hypothesised that the global energetic function of the foot would be analogous to a spring-damper system. The level of damping in such systems is typically proportional to velocity; as such, we also hypothesised that the fraction of energy dissipated by the foot, relative to that absorbed (foot-energy dissipation ratio) during the stance phase of running would increase with running speed.

## Results

Mean ± standard deviation and effect sizes for kinematic and foot energetic data are presented in Table 1. As running speed increased, ground contact time decreased (P = 0.01). The foot contacted the ground with the ankle in a relatively neutral orientation for all running speeds, with the ankle in slight dorsiflexion at the slower speed, moving to slight plantar flexion as running speed increased (P = 0.01).

**Table 1:** Mean ± standard deviation for kinematic and foot energetics data.

	2.2 ms^−1^	3.3 ms^−1^	4.4 ms^−1^	P-Value (Effect Size)		
				2.2 v 3.3	2.2 v 4.4	3.3 v 4.4
**Kinematics**						
Contact time (s)	0.27 ± 0.04	0.21 ± 0.02	0.17 ± 0.01	0.01 (1.9)	0.01 (3.4)	0.01 (2.5)
Ankle angle at foot contact (deg)	−2.0 ± 4.0	0.0 ± 4.0	3.0 ± 8.0	0.17 (0.5)	0.01 (0.8)	0.01 (0.5)
**Foot Energetics**						
Negative work (J.kg^−1^)	−0.23 ± 0.05	−0.33 ± 0.05	−0.44 ± 0.14	0.01 (2.0)	0.01 (2.0)	0.01 (1.0)
Peak negative power (W.kg^−1^)	−2.6 ± 0.7	−4.3 ± 1.1	−6.5 ± 1.7	0.01 (1.8)	0.01 (3.0)	0.01 (1.5)
Positive work (J.kg^−1^)	0.16 ± 0.06	0.17 ± 0.09	0.16 ± 0.10	0.99 (0.1)	0.99 (0.1)	0.99 (0.1)
Peak positive power (W.kg^−1^)	2.4 ± 0.7	3.5 ± 1.4	4.3 ± 2.2	0.01 (1.0)	0.02 (1.1)	0.26 (0.4)
Energy dissipation ratio	0.29 ± 0.2	0.48 ± 0.20	0.63 ± 0.22	0.01 (1.0)	0.01 (1.6)	0.01 (0.7)

**Figure 1:**
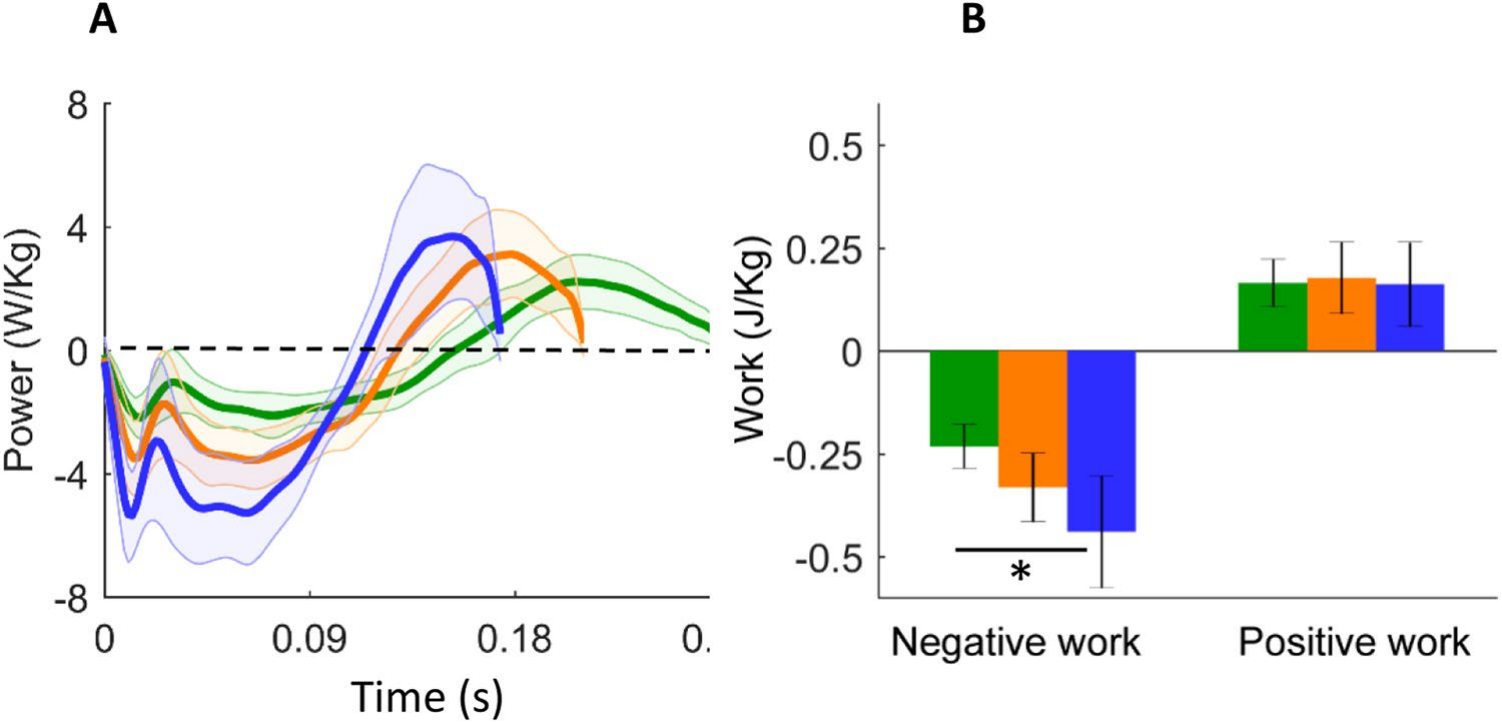
(**A**) Group mean ± SD foot power time series data calculated during stance when participants ran at 2.2 (green) 3.3 (orange) and 4.4 m.s^−1^ (blue). (**B**) Negative and positive work performed for deformable structures within the foot during the same running conditions described above. Negative work increases with running speed, while positive work remains relatively constant. Foot contact (FC) and toe off (TO). *Denotes significant difference between running speeds.

Figure 1A reveals that at all running velocities, the foot performed negative work from early stance through to mid-stance and subsequently performed positive work in late stance. The foot performed significantly more negative work with increasing running speed (P = 0.01) and did so at a greater rate (peak negative power, P = 0.01). The magnitude of positive worked performed by the foot remained constant across all running speeds (P = 0.67). There was a significant main effect of running speed on the rate of mechanical energy return/generation (peak positive power, P = 0.01). Post-hoc pairwise comparisons indicated that peak positive power was significantly higher when running at 3.3 m.s^−1^ and 4.4 m.s^−1^, compared to 2.2 m.s^−1^ (P = 0.01 and P = 0.02 respectively). However there was no difference in peak positive power between the 3.3 m.s^−1^ and 4.4 m.s^−1^ speeds (P = 0.26).

The foot energy dissipation ratio significantly increased with running speed (P = 0.01), primarily due to the increasing magnitude of negative work as participants ran faster. When running at 2.2 m.s^−1^ the foot behaved like a spring damper dissipating 29% of the energy absorbed during early stance. A greater proportion of absorbed mechanical energy was dissipated by the foot as running speed increased, dissipating 48% and 63% of the energy absorbed when running at 3.3 m.s^−1^ and 4.4 m.s^−1^, respectively.

## Discussion

This study provides unique insight into the energetic function of the human foot across a wide range of sub-maximal running speeds. When the combined contribution of all structures within the foot are considered, the foot appears to behave as a spring-damper, with an increasing proportion of absorbed mechanical energy being dissipated by the foot at higher running speeds.

Despite performing positive work and contributing mechanical energy to aid propulsion, the foot-energy dissipation ratio was greater than zero at all running speeds. This key finding suggests that the foot dissipates a substantial portion of the energy it absorbs, especially at higher running speeds. It seems counter-intuitive that such a large proportion of mechanical energy (~12 J) would be dissipated by the foot, as any energy dissipated will need to be generated by contracting muscles elsewhere in the body, in order to maintain net zero mechanical work at constant running speed^17–19^. A potential explanation may be that energy damping within the foot may slow centre of pressure progression, prolonging ground contact time and maximising the time in which a propulsive impulse can be generated. Thus, energy loss at the foot may be offset by greater metabolic energy savings created by larger, more proximal muscles producing force under more favourable force-velocity conditions^20,21^.

The foot is often referred to as a spring-like structure that stores and returns mechanical energy, providing considerable metabolic energy savings during running^1,3^. The positive work performed at the foot in the current experiment (~12 J) is in the range reported in previous experiments investigating energy storage and return from the plantar fascia (8–17 J). A key difference in the current data is the large magnitudes of negative work performed at the foot (~24 J), with approximately half of this energy being dissipated. Previous experiments have focussed on the contribution of the PF to energy storage and return within the foot^3,5,22^. Due to the elastic nature of the PF, presumably much of the absorbed energy is returned to the body via elastic recoil, with very little hysteresis^1,3,5,22^. The application of the unified deformable segment model in the current study allows quantification of the energetic contribution of all deformable structures acting at the foot, including but not limited to the PF. Thus, while it is apparent based on previous data, that the plantar fascia recycles mechanical energy, it appears that other structures within the foot act in parallel to dissipate energy.

The plantar fat pads of the heel and forefoot compress and dissipate energy in the period initially following foot-ground contact^7,10,11^. However the magnitude of energy absorbed by the fat pad of the heel *in-vivo*, has been estimated to be in the range of 2.5 joules^11^, considerably less than the 12 joules dissipated by the foot in the current experiment. An alternative explanation is that the foot behaves as an active spring-damper, with energy dissipated by active muscle lengthening at higher running speeds, when the loading rate of the foot is higher due to shorter ground contact time. The MTUs of the plantar intrinsic foot muscles are known to lengthen and shorten during stance phase with the muscles being active during this period. Therefore the intrinsic foot muscles have the potential to perform negative and positive work at the foot^4^. Furthermore, activation of these muscles increases with running speed^4^, providing support for the idea of the foot as an active spring-damper that dissipates a greater proportion of absorbed energy as loading rates increase. While this suggestion is biologically plausible, it is currently unknown whether the muscle fibres of the intrinsic foot muscles undergo lengthening during stance phase and further research is required to explore this hypothesis.

The discovery that foot behaves in a manner comparable to a viscous spring-damper system, has considerable implications for design of biologically inspired prostheses and wearable assistive devices (exoskeletons). For example, passive prosthetic feet designed for trans-tibial amputees are generally designed with spring-like properties to mimic the proposed function of the ankle and foot^23–25^. Further, wearable assistive devices (exoskeletons) are also designed to augment the function of the human ankle and foot, and have been shown to substantially reduce the metabolic cost of transport in healthy populations^26,27^. The incorporation of visco-elastic dampers in the feet of prosthetic and exoskeleton devices may further improve the interaction of these devices with humans. This improved function could potentially facilitate further reductions in the metabolic cost of transport for those who require these devices.

Participants generally landed with their ankle in a neutral orientation for all running speeds. A small but significant speed dependent alteration in ankle angle was observed, with a 5 degree increase in ankle joint plantar flexion from the slowest to fastest speed. It is possible that the change in ankle joint orientation at foot contact may have influenced the energetic function of the foot^5,28^. Based on our previous research exploring the influence of foot-strike pattern on the mechanical function of the LA^28^, it is possible that an increase in ankle plantar flexion at foot contact could lead to an increase in negative and positive work at the foot. However, due to the small magnitude of change in ankle angle observed in this study (5 degrees) compared to our previous work (approx. 20 degrees) we believe any change is likely to be minimal.

## Conclusion

The foot appears to behave as a viscous spring-damper during running. The underlying mechanism for how this occurs, the varied contributions of all the individual structures, and the subsequent mechanical and energetic consequences require further investigation.

## Methods

### Participants

Fourteen healthy male subjects (mean ± standard deviation for age 27 ± 5 years; height: 179 ± 6 cm; mass: 77 ± 9 kg) with no history of lower limb injury in the previous six months or known neurological impairment volunteered to participate in the study. Written informed consent was obtained from each subject. The study protocol was approved by The University of Queensland Human Research Ethics Committee and conducted in accordance with the Declaration of Helsinki.

### Experimental Procedures

Subjects performed barefoot running trials at 2.2 m.s^−1^ (slow), 3.3 m.s^−1^ (medium) and 4.4 m.s^−1^ (fast) on a force-instrumented treadmill (AMTI, force-sensing tandem treadmill, Watertown, MA, USA). To ensure familiarity with the treadmill and each running velocity, subjects were allowed 1 min to familiarise themselves to each speed, prior to the commencement of data capture. Kinetic and kinematic data were collected simultaneously during all running trials, with approximately 15–20 strides (foot contact to ipsilateral foot contact) being recorded at each running velocity for subsequent data analysis.

### Data Acquisition

#### Kinematic and kinetic measurements

Three-dimensional (3D) motion-capture of the foot and shank, and ground reaction force data were collected during each running trial. Retro-reflective markers (diameter 9.0 mm) were placed on the skin of the right foot in accordance with a previously described foot model, allowing modelling of a calcaneus segment and a shank segment^29^. Kinematic data was captured at 200 Hz using an eight camera 3D optoelectronic motion capture system (Qualysis, Gothenburg, Sweden) while kinetic data were synchronously captured at 2 kHz through an analogue to digital converter, using the Qualysis Track Management software (Qualysis, Gothenburg, Sweden).

### Data analysis

#### Data management

Kinematic and kinetic data files were exported to Visual3D (C-motion Inc., Germantown, MD, USA) for analysis. All marker data were digitally filtered with a 10 Hz low pass, recursive, second order Butterworth filter. Ground reaction force data were digitally filtered with a 35 Hz low pass, recursive, second order Butterworth filter. A vertical ground reaction force threshold of >50 N was set to define each foot contact, while toe-off was defined as occurring when vertical force was <50 N. Stance phase was defined as occurring between right foot contact and right toe-off. Assumed rigid segments were created for the shank and calcaneus according to a previously described multi segment foot model^29^. Ankle angle, defined as the rotation of the calcaneus segment relative to the shank segment, was calculated for all trials. The ankle angle during the running trials was offset to the ankle angle calculated during a static standing trial, such that a 0° ankle angle was indicative of the ankle angle during the quiet standing position. Negative values indicate dorsiflexion of the ankle. Mean ankle angle at foot contact was calculated for each individual at each running speed, by averaging the ankle angle at foot contact for each stride cycle during the respective trial.

#### Foot power analysis

We applied a unified deformable (UD) segment analysis to quantify the instantaneous power of the foot as it interacts with the ground^23,30^. This approach (described in detailed elsewhere^23,30^), models the foot as a hybrid segment with a proximal rigid component (calcaneus) and an in-series deformable distal component. In brief, when structures such as the plantar fat pads and longitudinal arch deform, or the metatarso-phalangeal joints extend or flex, there will be an associated displacement of the location of the ground reaction force (centre of pressure (COP)) relative to the centre of mass (COM) of the rigid proximal component of the segment. The total deformation velocity of the deformable component of the foot (V_foot_) can be quantified by accounting for the rotational and translational velocities of the proximal rigid component (Equation 1).

1 $${{\rm{V}}}_{{\rm{foot}}}={{\rm{V}}}_{\mathrm{cm}\_\mathrm{calc}}+({{\rm{W}}}_{{\rm{calc}}}{\rm{X}}\,{{\rm{r}}}_{\mathrm{calc}\_\mathrm{COP}})$$

Where V_cm_calc_ represents the translational velocity of the calcaneus segment, W_calc_ represents the rotational velocity of the calcaneus segment and r_calc_COP_ represents the distance from the COP to the calcaneus COM.

Subsequently foot power (P_foot_) can be quantified by summing the dot product of foot deformation velocity (V_foot_) and GRF (F_GRF_) with the dot product of the free moment (M_free_) and angular velocity of the calcaneus (W_calc_), as represented in Equation 2.

2 $${{\rm{P}}}_{{\rm{foot}}}=({{\rm{F}}}_{{\rm{GRF}}}\cdot {{\rm{V}}}_{{\rm{foot}}})+({{\rm{M}}}_{{\rm{free}}}\cdot {{\rm{W}}}_{{\rm{calc}}})$$

Thus, foot power reflects the rate of energy added or removed from the body via structures distal to the calcaneus segment (Fig. 1).

Energy added (positive work) and/or removed (negative work) from the body by structures within the deformable foot segment was calculated during stance via trapezoidal integration of the foot segment power. The peak rate of energy absorption (peak negative foot power) and peak rate of energy return/generation (peak positive foot power) were also calculated. Foot-energy dissipation ratio, defined as the net work performed by the foot during stance phase, divided by the total negative work, was calculated to determine the relative proportion of mechanical energy dissipated during each foot contact. A foot-energy dissipation ratio of one is indicative of a pure damper, with every joule of energy absorbed, being dissipated by structures within the foot. For each individual, negative and positive work performed about the foot, peak negative foot power, peak positive foot power and foot energy dissipation ratio were calculated during stance phase. Data was analysed across a minimum of 15 stride cycles for each running velocity and normalised to body mass. This data was subsequently averaged to form a participant mean for each variable, at each gait velocity. The data presented within Fig. 1 is produced using the same procedure, with individual mean foot-power time-series data averaged across the group to create a time-series ensemble average at each running speed.

#### Statistics

A one-way repeated measures analysis of variance (ANOVA) was used to describe the effect of running speed on mechanical energy absorbed (negative work) and returned/generated (positive work), peak rate of energy absorption (peak negative foot power) and peak rate of energy return/generation (peak positive foot power) and foot-work ratio. Post-hoc multiple comparison tests, including Bonferroni corrections, were performed between each running velocity (2.22 v 3.33 v 4.44 m.s^−1^). Statistical differences were established at P ≤ 0.05. Effect sizes (ES) for post-hoc comparisons are presented as standardized mean differences and classified as trivial (ES < 0.2), small (ES = 0.2–0.3), moderate (ES = 0.3–0.7) and large (ES > 0.7). Results are presented as mean ± standard deviation (SD) unless otherwise stated.

### Data availability

The datasets generated during and/or analysed during the current study are available from the corresponding author on reasonable request.
